# The Importance of Clinical Data for the Diagnosis of Breast Tumours in North Afghanistan

**DOI:** 10.1155/2021/6625239

**Published:** 2021-07-30

**Authors:** Gerhard Stauch, Peter Fritz, Rauofi Rokai, Atiq Sediqi, Haroon Firooz, Hans Ullrich Voelker, Michael Weinhara, Joachim Mollin, Bishara Soudah, Peter Dalquen, Friedhelm Brinckmann, Jürgen Dippon

**Affiliations:** ^1^Department of Pathology, Aurich, Westerstede, Germany; ^2^iPath Telemedicine Network Gemeinnützige GmbH, D-26603 Aurich, Germany; ^3^Robert Bosch Hospital, Department of Pathology, Stuttgart, Germany; ^4^Abu Ali Sina Hospital, Department of Pathology, Masar E Sharif, Afghanistan; ^5^Haroon Firooz Medical Laboratory, Herat, Afghanistan; ^6^Leopoldina Hospital, Department of Pathology, Schweinfurt, Germany; ^7^Epos/Gopa GmbH, Frankfurt /M, Germany; ^8^myCare2x Netzwerk, Healthcare Consulting GmbH, Ebersberg, Germany; ^9^University MH Hannover, Institute of Pathology, Hannover, Germany; ^10^University Basel, Institute of Cytology, Basel, Switzerland; ^11^Onkologischer Schwerpunkt Stuttgart, Stuttgart, Germany; ^12^University of Stuttgart, Institut für Stochastik und Anwendung, Fachbereich Mathematik, Stuttgart, Germany

## Abstract

**Background:**

This study was performed in knowledge of the increasing gap between breast disease treatment in countries with restricted resources and developed countries with increasingly sophisticated examination methods.

**Methods:**

The authors present the analysis of a breast disease register consisting of diagnostic cases from Mazar e Sharif and Herat in 2018 and 2019. The study comprises a total of 567 cases, which were presented to experts via telemedicine for final diagnosis. 62 cases (10.9%) were excluded due to inacceptable data or insufficient image quality. These data provided by daily diagnostic classification were used for the built-up of a profile for each frequent breast disease and a breast cancer register. All images and cases were seen by at least 3 independent experts. The diagnoses were made in 60% of cases by cytology of fine needle aspiration and in 40% by histological images.

**Results:**

For each entity of breast diseases (e.g., fibroadenoma), a profile of context variables was constructed allowing to assist medical decisions, as “wait and see,” elective surgery or immediate surgical intervention with R0 (complete) resection. These “profiles” could be described for fibroadenoma, mastitis, galactocele, fibrous-cystic disease, and invasive breast cancer.

**Conclusions:**

The presented preliminary data set could serve as a cost-effective basis for a North Afghan breast cancer registry, with option to extent to a national model. These preliminary data are transformed in profiles of breast diseases, which are used by the local physicians in charge of breast disease patients. Each new case can be compared by the local treating physician with the profile of all preceded cases with the same diagnosis.

## 1. Introduction

The topic of breast cancer highlights the problem of delayed diagnostic and therapy of Noncommunicable Diseases (NCD) in middle- and low-income countries (MLIC). The indicators of weak medical infrastructures in low-income countries [1, 2] are delayed time to diagnosis and a higher frequency of advanced breast cancer. This applies particular in Afghanistan, with a health system in disarray for many years due to ongoing conflicts and violence. The reconstruction of the health system in Afghanistan is currently being tackled by numerous groups, including the Afghan and foreign governments and several nongovernmental organizations (NGOs). Some progress has been made, but there are still many hurdles, including a shortage of experienced doctors and medical staff, especially in rural areas where 74% of the population lives. In these areas, patients suffer from poor hygiene, poor transport facilities to health centres and limited medical knowledge in self-assessment [3–6]. There are only sparse data on breast cancer in Afghanistan [7–9].

In 2019, the first national symposium on breast cancer was held in Afghanistan. On this occasion, participants from the Afghan NGOs and the Ministry of Public Health (MoPH) reported about 30,000–60,000 patients per year [10, 11]. Since the data are differing widely, doubts about the validity of these figures are justified. One way to improve the unsatisfactory diagnostic situation is to combine daily diagnosis of breast diseases with a breast disease register allowing to build up a local profile for each breast disease. This supports even unexperienced physicians in diagnoses and treatment of breast diseases.

One step towards improving the local situation was the establishment of two pathology departments in Masar e Sharif: in 2010 at the referral Hospital Abu Ali Sina and in 2016 at the Family Health Hospital. Both provide histological and cytological diagnostics for 1.5 million people in Northeast Afghanistan. These departments have been linked to a third centre in Herat, managed by another NGO, which serves about 2 million people. These three departments regularly send digitized microscopic images of histological and cytological preparations and the related clinical information of the patients to European experts via the telemedicine service iPath-Network [12–14]. Up to now, more than 14,500 cases have been diagnosed or confirmed. This opens up new possibilities to use the data for development of an artificial intelligence-based system to support and control morphological diagnostics.

### 1.1. Aim of the Study

This study presents the profile of breast disease in Northern Afghanistan and evaluates the prevalence of inflammatory, benign and malignant diseases in the area of Masar E Sharif and Herat with the aim (1) to provide an overview of the situation of breast patients, (2) to collect data for the further development of disease profiles, (3) to sensitize political decision makers to this special medical problem and enable low cost diagnostic and treatment decisions, and (4) to establish a local register for breast diseases.

## 2. Materials and Methods

### 2.1. Patients

Between January 1^st^ 2018 and December 31^th^ 2019, 3800 cases of three Departments of Pathology in North-Afghanistan were classified via the iPath telemedicine network by four European pathologists (BS, GS, PD, and PF). Of these cases, 567 were related to breast disease, of which 62 (10.9%) were excluded from the study for the following reasons: (1) male gender, (2) sampling errors, (3) inappropriate digital images, (4) rare diagnoses such as malignant lymphomas, and (5) lack of basic clinical data as gender or localization.

This left a total of 505 cases for evaluation. The microscopic photos were taken at magnification 100 and 400 times and converted into JPEG-images. The histological preparations were stained with Haematoxylin & Eosin (H&E), the cytological ones with Papanicolaou's and H&E technique. In 20% of cases, macroscopic images were added to the microscopic set.

Ultrasound or mammographic images were available in less than 3%. Analysis of estrogen-receptor (ER) and progesterone-receptor (PR) as well as Her2/neu-receptor was not available. All cases were identified by an ID-numbers of the local Afghan pathology departments and by an ID-numbers of iPath telemedicine network [13].

### 2.2. Context Variables

The following variables were reported by the local physician in charge when submitting a case for expert consultation on the iPath-Network: (1) age in years; (2) duration of disease in months; (3) size in mm determined by palpation; (4) consistency: soft, firm, or absent information (NA); (5) tumour margin: regular, irregular, or NA; (6) tumour mobility: mobile, fixed, or NA; (7) skin involvement: yes, no, or NA; (8) axillary involvement: yes, no, or NA; (9) pain: painful, painless, or NA; (10) lactation/late pregnancy: yes, no, or NA; and (11) birth history: yes, no, or NA.

### 2.3. Formation of Diagnosis

In all cases, at least three pathologists have made a diagnosis. In case of discordance, a final diagnosis was made after discussion with consensus. The diagnoses were classified in terms of WHO Classification (5th edition 2018) [15] or ICD-10 and ICD-O-3 system [16, 17] with some additional remarks such as grading of the carcinoma. In each case, the type of preparation, histological or cytological, was noted. The diagnostic data were either numerical or categorical.

### 2.4. Comparison with Selected Breast Cancer Data Set

For demonstration of the significance of the local tumour data set, we compared the data with the data of the OSP (Onkologischer Schwerpunkt Stuttgart) [18, 19] and TGCA data set (Cancer Genome Atlas) [20]. The OSP breast cancer register consists of approximately 30,000 breast cancer patients from the year 1990 up to 2020. The TCGA data set consisted of 1,089 breast cancer patients. This comparison was done for identifying special features of Afghan breast cancer patients.

### 2.5. Statistic

All data were exported from iPath-Network (Afghanistan-project) and transferred in a table calculation data set (Excel, Microsoft). This data set was analysed in R (R project for statistical computing version 3.5.3) [21]. Statistical significance was assumed for *p* < 0.05. A result was designed highly significant if *p* < 0.00001. All context variables were considered as meaningful for decision-making. The possible decisions were benign or malignant defining an invasive breast cancer (IBC). All missing values were eliminated by applying mouse package (R package) for replacing NA values [22] (attributes: default, *m* = 5). With the result of the mouse package replacing the missing values, we built up a profile for each frequent diagnosis in breast diseases (Tables 1–6).

### 2.6. Ethics

All cases were completely and irreversibly anonymized, and for each case, neither the patient's name nor birthday was known. Each case was identified by an alpha-numeric code given without name or birth date. No financial interest of the authors exists.

## 3. Results

### 3.1. Case Classification

Out of the 505 cases 366 (72.5%) were classified as benign and 139 (27.5%) as malignant. Most diagnoses (322 cases corresponding to 63.7%) were made cytologically, compared with 183 (36.3%) diagnosed by a histological examination. With 245 of 366 (66.9%), benign lesions were diagnosed significantly more frequently on cytological specimens compared to 121 (33.1%) on histological specimens. Conversely, malignant tumours were only slightly more frequently diagnosed on cytological specimens with 77 of 139 (55.4%) compared with 62 (44.6%) diagnosed on histological specimens. This difference is significant (*p* = 0.02) (see Table 7).

### 3.2. Context Variables

An univariate analysis shows that, on average, almost all context variables of benign and malignant breast disease differ, especially with regard to age, tumour margin, skin involvement, axillary involvement, mobility, and lactation (Table 6). However, this could only be demonstrated after using the R-package of MICE [21], which allows elimination of missing values. We provided therefore an Excel data set with a k∗n table (505 rows∗19 columns), from which each breast order profile could be extracted (Table 1).

### 3.3. Benign Breast Diseases: Fibroadenoma

**Figure 1:**
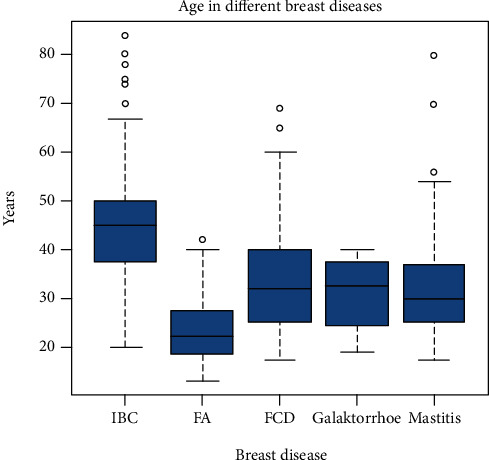
Age distribution in North Afghanistan breast diseases. IBC: invasive breast cancer: FA: fibroadenoma; FCD: fibrocystic diseases. Circles signify outliner, and the horizontal labels are the range without outliners. The blue boxes signify the 25 to 75 percentile.

147 cases were classified as fibroadenoma (FA). A profile of these 147 cases was settled allowing each local physician to see how well a new individual case fits the context variables (Table 2). Not painful as well as movable and firm consistency in a breast mass of a young patient are the hallmarks of a FA. If the context variables and the morphological findings (mostly cytological ones) are in favour, the local physicians will be recommended to do elective surgery and to avoid antibiotic therapy. As shown in Figure 1, there are hardly any statistical outliers in the group of FA as compared to the high numbers in IBC (see Figure 1).

### 3.4. Mastitis

Mastitis (Table 3) was often described as a breast masse with irregular margins, skin involvement pain, and correlated with the history of lactation. If these context variables are confirmed in a patient, the favoured decision is treatment with antibiotics and incision.

### 3.5. Fibrocystic Disease

In 73 cases (Table 4), we diagnosed a fibrocystic disease The tumour-like appearance of *fibrocystic change* correlated with a low percentage of axillary involvement (8.2%), firm consistency (90.4%), and an intermediate age (32.6 years, SD = 11.0).

### 3.6. Galactocele

*Galactoceles* (Table 5) were characterized by a low percentage of axillary lymph node involvement (14.7%), a firm consistency (83.3%), and lactational status (87%). All galactoceles were diagnosed by FNA.

### 3.7. Malignant Breast Diseases

**Figure 2:**
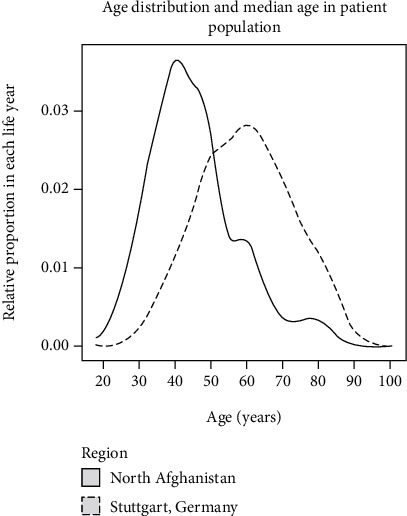
Age distribution in breast cancer patients in Afghanistan and Germany.

*Invasive breast carcinomas* (Table 6) were mostly classified as ductal invasive carcinomas mostly without subtyping (IBC NST) or grading. Only 3 IBC were classified as lobular breast cancer (LIC). As shown in Table 6, an irregular margin (93.9%) and firm consistency (93.9%) were the main features of the IBC. Skin and axillary involvement were found in 82.7% and 68.4%, respectively, of the IBC cases. The main characteristic of the IBC, which distinguished it from all benign breast diseases, was that the patient's mean age was higher than all other disease groups (mean age = 45.5 years, SD = 12.2) (Figures 1 and 2, Table 6). The knowledge of the presence of the suspicious context variables enabled the local physician to avoid unnecessary treatment options and to recommend surgical excision either lumpectomy or mastectomy and adjuvant hormone and chemotherapy.

### 3.8. Comparison of the Data of Afghan Breast Cancer Patients with Two Breast Cancer Data Sets [18–20]

Three interesting differences with consequences for treatment decisions could be demonstrated: (1) breast cancer patients in Northern Afghanistan are about 14.4 years younger than German breast cancer patients (mean = 45.4, SD = 12.42; median = 45, range: 19-84, versus mean = 59.8, SD = 13.24, median = 59.6, range 18.2-100.4, *p* < <0.000001); (2) the frequency of breast cancer patients under 30 years of age was 10 times higher in Afghanistan than in Germany (7/139 corresponding to 5% versus 76/16321 corresponding to 0.47%, *p* < <0.00001); and (3) Afghan patients are significantly more likely to have advanced stage pT3 and pT4 breast carcinomas at the time of diagnosis, corresponding to a size > 5 cm (36/139 = 25.9% versus *N* = 2060/16330 = 13.1%, *p* = 0.000005). TGCA data (Cancer Genome Atlas) [20] were in line with the OSP data (Onkologischer Schwerpunkt Stuttgart) [18, 19] giving a mean age of 58.5 years (SD = 13.2, mean: 58 years). In a similar way, TGCA data [19] yield a frequency of pT3 or pT4 breast cancer of 16.9%, comparable with the OSP data. This comparison highlights the specifics of breast cancer in North Afghanistan and the need for improvement of patient management.

Using iPath-Network for giving a final diagnosis to breast diseases diagnosed either FNA (fine needle aspiration), histological approach CNB (core needle biopsy), or SB (surgical biopsy) was free of technical problems except some internet interruption. Language problems were not dominant as far as all participants were speaking English. Giving the diagnosis as ICD-O or ICD-10 code allows an easy transformation to Farsi which is equivalent to the national language Dari. This is initiated by a tumour register in which ICD-10 and ICD-O codes are used for interlingual communication. All these data are communicated in the iPath-Network platform [12, 13].

## 4. Discussion

A “profile” of the most frequently observed breast diseases such as fibroadenoma, mastitis, fibrocystic disease, galactocele, and invasive breast cancer (IBC) was provided within the frame of this study (Tables 2–6). Selected context variables were assigned to each disease profile. It enables the Afghan doctor responsible for the patient to assess whether the clinical or morphological diagnosis fits the patient's disease. For example, the probability that a circumscribed breast mass of a young woman is a fibroadenoma and not a malignant change is greater (95%). The results of the study may not only support diagnosis and confirm the plausibility of a diagnosis and treatment decision but may also be relevant to public health decisions and statistical data validation.

The prevalence of malignant tumours was higher in this study with 139 cases (27.5%) than in comparable studies from North Africa, the Middle East [23, 24], Europe [25, 26], or Iran [27]. This phenomenon can be explained by the specialization of the authors RR and AS in this field and the increased awareness of the risk population in the region. The latter was the result of a consistent sensitization of the risk population through repeated training of nurses, midwives, and general practitioners in Mazar e Sharif.

Most of the benign breast diseases showed characteristics in the context variables that distinguish them from the IBC patient group. Fibroadenomas show a clear prevalence towards younger age. This finding is consistent with the findings of other authors [26, 28]. It seems that younger women use more self-examination techniques and increased attention to their breasts, which may have an impact on the detection of lesions in risk groups. However, this phenomenon is limited to the urban population and cannot be detected in patients from rural areas (Dr. Rokai personal communication). It could be the result of training programs for nurses and midwives started by Dr. Rauofi Rokai in 2016. Mastitis has been associated with lactation in 85.7% (Table 2) of cases, most likely a cause that this rate is higher as in the neighbouring countries [23–25, 27, 29, 30]. The reasons may be a long breast feeding time and inadequate hygiene measurements. Both diagnoses, fibroadenoma and mastitis, were mostly made by FNA. The advantage of this procedure over histological diagnosis is low cost and faster diagnosis. The disadvantage of the FNA approach, the need for a high level of experience for a final diagnosis, is overcome by the telemedical diagnosis of each individual case by pathologists experienced in cytology (PD, BS, GS, and PF) and can be supported by histological techniques like CNB (core needle biopsy) and/or open biopsy.

From our available data, some interesting epidemiological features can be identified, as the comparison of the North Afghan data set with its German counterpart shows. Afghan breast cancer patients come to the initial examination with a more advanced tumour stage and are on average about 14 years younger, whereby the higher proportion of patients under the age of 30 is particularly striking. The younger age of Afghan breast cancer patients can be explained by a special type of breast cancer (usually triple negative), a shorter life expectancy, and a difference in reproductive lifestyle. In contrast, 25.9% of patients in Afghanistan were in stage III or IV as compared to 13.9% in the German counterpart. The proportion of young patients under 30 years of age was also higher in the Afghanistan data set (5% compared to 0.5%) than in the German cancer registry. Both differences are significant. There is a great lack of publications about breast cancer in young women in countries with restricted resources as recognized by Galvez-Hernandez and coworkers in 2017 [30]. A breast cancer register may be a first step in better treatment for such patients in countries with restricted resources. For this approach, methods of computational statistics as the mouse package in R are mandatory [21].

The presented study could form the basis for a disease register of female breast tumours in Northern Afghanistan with continuous improvement. Each new case of breast disease is entered into the described data register and continuously improves the recognition characteristics of each disease entity. Reporting each diagnosis also as ICD-10 code or ICD-O-3 code [15, 16] opens the possibility to an automatic transformation to FARSI.

Finally, the disease registry should be supplemented by an annual follow-up at least in IBC patients in order to be able to examine diagnostic and therapeutic measurements in view of Overall Survival (OVS) and Disease-Free Survival (DFS). In summary, our data are useful in each case of breast disease and help to improve treatment by collecting knowledge from each single case and to diminish the growing gap between high-income countries with sophisticated research methods [31–33] and low-income countries with lack of nearly all modern technologies like molecular biology or immunohistochemistry [30].

## Figures and Tables

**Table 1:** Context variables and correlation in benign lesions versus invasive breast cancer.

Variable	Benign cases *N* = 366 (%)	IBC *N* = 139 (%)	Correlation with DIC *t*-value or chi^2^, *p*	Percentages of missing values *N* (%)^∗^
Age (years)			14.5	5
Mean	28.5	45.5	*p* < <0.000001	1.0
SD	10.2	12.2		
Median	26	45		
Size (cm)			3.03	120
Mean	2.6	3.9	*p* = 0.0021	(23)
SD	2.0	3.1		
Median	3-0	3.0		
Pain			7.98	159
No	192 (52.5)	10 (71.9)	*p* = 0.0047	(30.9)
Yes	144 (47.5)	39 (28.1)		
Margin			40.6	279
Regular	140 (38.2)	12 (8.7)	*p* < 0.00001	(54.2)
Irregular	226 (61.8)	127 (91.3)		
Movability			48.5	420
No	164 (44.8)	111 (75.9)	*p* < <0.000001	(81.7)
Yes	202 (55.2)	28 (20.1)		
Skin involvement			(88.6)	351
No	234 (63.9)	23 (16.6)	*p* > >0.00001	(68.3)
Yes	132 (36.1)	116 (83.4)		
Children			28.9	483 (94.0)
No	162 (44.3)	100 (71.9)	*p* < 0.000001	
Yes	204 (55.7)	39 (28.1)		
Lactation			25.4	387 (75.3)
No	119 (32.5)	80 (57.6)	*p* = 0.0000004	
Yes	247 (67.5)	59 (42.4)		
Axillary involvement			147.7	391 (76.1)
No	336 (91.2)	57 (41)	*p* < <0.000001	
Yes	30 (8.2)	82 (59)		
Consistency (firm)			4.8	291 (57.6)
No	48 (13.1)	8 (5.8)	*p* = 0.028	
Yes	318 (86.9)	131 (94.2)		
Duration (months)			55.7	63 (12.3)
Mean	10.42	18.04	*p* = 0.00025	
SD	10-0	29.3	(Kruskal test)	
Median	5	10		

^∗^Note that the missing values are replaced by the mice soft package of R.

**(a):** 

Profile of fibroadenoma (*N* = 147)				
	Mean	SD	Median	Range
Age (years)	23.6	6.6	22	12-42
Duration of disease (months)	11.0	17-6	6	1-26
Size (cm)	2.6	1.7	2.0	1-15

**(b):** 

Categorical variables				
	No (*N*)	Yes (*N*)	No (%)	Yes (%)
Painful	98	49	66.7	33.3
Movability	43	104	29.3	70.7
Firm consistency	8	139	5.8	94.2
Irregular margin (regular)	84	63	57.1	42.9
Skin involvement	119	28	81	19
Axillary involvement	137	10	93.2	6.8
Child	70	77	47.6	52.4
Lactation	63	84	42.9	97.1

**(a):** 

Profile of mastitis (*N* = 77)				
	Mean	SD	Median	Range
Age (years)	32.1	10.7	30	17-80
Duration of disease (months)	4.1	6	2	0-36
Size (cm)	3.4	1.5	3.0	1-12

**(b):** 

Categorical variables				
	No (*N*)	Yes (*N*)	No (%)	Yes (%)
Painful	22	55	28.6	71.4
Movability	51	26	66.2	33.8
Firm consistency	22	55	28.6	71.4
Irregular margin	17	60	22.1	77.9
Skin involvement	30	47	39	61
Axillary involvement	60	17	77.9	22.1
Child	16	61	20.8	79.2
Lactation	15	62	19.5	80.5

**(a):** 

Profile of fibrous-cystic breast disease (*N* = 73)				
	Mean	SD	Median	Range
Age (years)	32.6	11.0	32	17-69
Duration of disease (months)	12.4	20.4	6	1-120
Size (mm)	3.0	1.5	30.0	6-70

**(b):** 

Categorical variables				
	No (*N*)	Yes (*N*)	No (%)	Yes (%)
Painful	35	38	48	62
Movability	43	30	58.9	41.1
Firm consistency	7	66	9.6	90.4
Irregular margin	25	48	34.3	65.7
Skin involvement	45	28	61.6	38.4
Axillary involvement	67	6	91.8	8.2
Child	28	45	38.4	61.6
Lactation	31	42	42.5	67.5

**(a):** 

Profile of galactoceles (*N* = 12)				
	Mean	SD	Median	Range
Age (years)	32.5	7.7	31.1	19-40
Duration of disease (months)	7	7.8	7.8	0.1-24
Size (mm)	2.3	1.1	2.0	10-100

**(b):** 

Categorical variables				
	No (*N*)	Yes (*N*)	No (%)	Yes (%)
Painful	5	7	41.7	58.3
Movability	7	5	58.3	41.7
Firm consistency	2	10	16.7	83.3
Irregular margin	5	7	41.7	58.3
Skin involvement	6	6	50	50
Axillary involvement	10	2	85.3	16.7
Child	4	8	33.3	66.7
Lactation	5	7	41.7	58.3

**(a):** 

Profile of invasive breast carcinoma (*N* = 139)				
	Mean	SD	Median	Range
Age (years)	45.6	12.2	45	20-84
Duration of disease (months)	17	10.0	12	0.3-144
Size (cm)	3.7	2.5	3	4-20

**(b):** 

Categorical variables				
	No (*N*)	Yes (*N*)	No (%)	Yes (%)
Painful	100	39	71.9	28.1
Movability	105	34	78.4	21.6
Firm consistency	8	131	6.1	93-9
Irregular margin	8	131	6.1	93.9
Skin involvement	24	115	17.3	82.7
Axillary involvement	44	95	31.6	68.4
Child	124	15	89.2	10.8
Lactation	50	89	36	64

**Table 7:** Incidence of benign lesions and malignant breast tumours.

Diagnosis	*N* and %	Diagnosis done by cytology	ICD-10	ICD-O
*Inflammatory breast diseases*				
Fibrous-cystic breast disease	73 14.5%	44/73 (60.3%)	N60.1	None
Mastitis	77 15.2%	59/77 (80.8%)	N61	None
Galactorrhoe	12 2.4%	12/12 (100%)	N64.8	None
Fat necrosis	6 1.2%	3/6 (50%)	M78.89	None
*Benign breast tumour*				
Adenoma	21 4.2%	4/21 (19.0%)	D24	8211/0, 8204/0, 8204/0
Fibroadenoma	147 29.1%	115/147 (78.2%)	D24	9010/0
Papilloma	8 1.6%	6/8 (75%)	D36.9, D24X	8503/0
Phyllode tumour	7 1.4%	1/7 (14.3%)	D24	9020/0, 9020/1
Radial scar	3 0.6%	0/3 (0%)	N60.2	None
Sclerosing adenosis	12 2.4%	1/12 (8.3%)	Not given	None
*Invasive breast carcinoma (IBC)*				
IBC	139 27.4%	77/139 (55.4%)	8500/3, 8520/3 or other specialized forms	C50.9

## Data Availability

All data are included within the text.
